# Supplementary material for: Acceptance of a Nordic, Protein-Reduced Diet for Young Children during Complementary Feeding—A Randomized Controlled Trial
Source: Foods. 2021 Jan 29;10(2):275. doi: 10.3390/foods10020275 (PMC7911089; doi:10.3390/foods10020275)
Supplement: Supplementary file 1 [file foods-10-00275-s001.pdf]

Table S1. Sensory properties of baby food purées evaluated by the sensory panel of adult participants.

	Sensory property	Reference sample	Concentration	Value in scale
**Aroma**	Total intensity of aroma<br>Sweet aroma<br>Sour aroma<br>Fresh aroma			
**Texture**	Grainy	Piltti baby food purée banana and lingonberry (Suomen Nestlé Oy, Finland)		2
		Rye lingonberry porridge (Saarioinen Oy, Finland)		6
	Watery	Piltti baby food purée banana and peach (Suomen Nestlé Oy, Finland)		2
		Piltti baby food purée apple and banana (Suomen Nestlé Oy, Finland)		6
	Thick	Apple purée (Bonne Juomat Oy, Finland)		3
	Mouth filling	Semper solskensfrukt och yoghurt (Semper Ab, Sweden)		6
	Sticky	Piltti baby food purée banana and peach (Suomen Nestlé Oy, Finland)		4
		Semper baby food purée solskensfrukt och yoghurt (Semper Ab, Sweden)		7
**Taste and flavor**	Total intensity of flavor			
	Sweetness	Sucrose soluation	1 %	2
		Sucrose solution	2,00 %	8
	Sourness	Citric acid solution	0,035 %	2
		Citric acid solution	0,07 %	6
	Bitterness	Caffeine solution	0,0175 %	2
		Caffeine solution	0,035 %	6
	Umami	Natrium glutamate solution	0,10 %	7
	Saltiness	Natrium chloride solution	0,05 %	8
	Astringency	Aluminum ammoniumsulfate solution	0,05 %	4
	Intensity of aftertaste			
